# Acculturative Stress of Chinese Rural-To-Urban Migrant Workers: A Qualitative Study

**DOI:** 10.1371/journal.pone.0157530

**Published:** 2016-06-14

**Authors:** Bao-Liang Zhong, Tie-Bang Liu, Jian-Xing Huang, Helene H. Fung, Sandra S. M. Chan, Yeates Conwell, Helen F. K. Chiu

**Affiliations:** 1 Department of Psychiatry, The Chinese University of Hong Kong, Hong Kong SAR, P. R. China; 2 Affiliated Mental Health Center, Tongji Medical College of Huazhong University of Science and Technology, Wuhan, P. R. China; 3 Shenzhen Key Laboratory for Psychological Healthcare, Shenzhen Institute of Mental Health, Shenzhen Kangning Hospital, Shenzhen Mental Health Center, Shenzhen, P. R. China; 4 Centre for the Study of Religion and Chinese Society, The Chinese University of Hong Kong, Hong Kong SAR, P. R. China; 5 College of Social Development, Fujian Normal University, Fuzhou, P. R. China; 6 Department of Psychology, The Chinese University of Hong Kong, Hong Kong, P. R. China; 7 Department of Psychiatry, University of Rochester Medical Center, Rochester, NY, United States of America; Federal University of Rio de Janeiro, BRAZIL

## Abstract

**Background:**

Global literature has suggested a negative impact of acculturative stress on both physical and mental health among international migrants. In China, approximately 20 percent of its population is rural-to-urban migrant workers and there are significant cultural differences between rural and urban societies, but no data are available regarding the acculturative stress of Chinese migrant workers. This study aimed to explore the forms and contexts of acculturative stress among Chinese migrant workers.

**Methods:**

Qualitative data were collected from four focus group discussions with 17 Chinese rural-to-urban migrant workers and three individual interviews with three medical professionals who provided mental health services for factory-workers in Shenzhen, China.

**Results:**

The data in the current study showed that rural-to-urban migrant workers in China had experienced various forms of acculturative stress including difficulties in adapting to the environment, work-related stress, family-related stress, financial hardship, and lack of sense of belonging to cities.

**Conclusion:**

Rural-to-urban migration in China is a challenging transition with significant acculturative stress and demands for major adjustments among migrant workers. The assessment and management of acculturative stress is a necessary first step in providing mental health services to migrant workers.

## Introduction

In China, the process of rapid urbanization and industrialization has resulted in massive work-related rural-to-urban migration over the last three decades. Rural-to-urban migrants constitute a special social entity, as they are officially rural residents according to the Chinese household registration system (***hukou***), owning farmland due to the land contract system, while adopting non-agricultural work and living on wages in cities without the social security coverage enjoyed by other urban residents[1]. According to the Chinese government statistics[2], in 2014, almost one out of every five Chinese– 274 million people–were rural-to-urban migrant workers and the total number of migrant workers is still increasing. These internal rural-to-urban migrants, despite their indisputable contribution to the rapid economic development in China, are still socially and economically disadvantaged, partly because of their rural identity, “floating” status and marginalized life in cities[3,4]. Numerous studies have revealed that Chinese internal migrants were at elevated risk for mental health problems[5–7], but the underlying causes for poor mental health remained unclear.

The global literature has suggested an association of cross-national migration with risk for poor health status both physically and mentally[8–10]. There are many personal and social-cultural factors (e.g. gender, social support, racism, unemployment, and cultural distance) that could contribute to such negative association[11,12]. One of the most frequently reported factors is the acculturative stress of immigrants in settlement countries[13]. Previous studies suggested that stress derived from cross-cultural adaptation may result in negative psychological changes among acculturating migrants and further lead to greater risk for depression, anxiety, and suicidal ideation[11, 14–22]. In addition, previous research has found that acculturative stress has an adverse effect on psychological well-being, quality of life and life satisfaction[23–25].

Though the adverse effect of acculturative stress on mental health is a universal phenomenon, the forms and nature of acculturation and acculturative stress may be specific to the immediate context of social system, culture and population characteristics. The bulk of international studies have shed lights on acculturation in the U.S. and Europe[26–28], but few focused on rural-to-urban acculturation within one nation. Due to the notable socio-cultural discrepancies between Western and Chinese contexts and differences between transnational and internal migration, the findings of Western studies on acculturative stress may not be generalizable to Chinese rural-to-urban migrant workers. Developing an effective intervention or public policy to reduce the negative impact of acculturative stress in a defined migrant population within a sociocultural context requires good understanding of the forms and context of acculturative stress in that particular population or culture.

### Theoretical Framework of Rural-to-Urban Acculturative Stress

China has seen enormous urbanization from a long-standing agricultural society in the past few decades and yet the rural population and workforce in the agricultural sector remain sizeable. In 2014, 618.7 million Chinese, or 45.2% of its total population, are employed in agriculture[29]. Since 1958, the Chinese government has introduced a national policy, namely the urban-rural dual structure[30], a system based on residential registration, to restrict the rural-to-urban migration of rural labor force. Due to the administrative barriers of this system, the cross-cultural and economic interactions between rural and urban areas are limited, perpetuating the rural-urban differences in socioeconomic and cultural milieu. Following the rapid industrialization in urban areas in recent years, the urban culture with industry civilization characteristics has been well-established in thriving coastal cities and concentrated industrial zones. Owing to the uneven economic development and other historical reasons, the urban-rural dual structure has resulted in China’s unique culture model: agricultural and industrial cultures coexist independently in its rural and urban areas[31]. As suggested by Li, the cultural difference between rural and urban regions, is the most representative cultural discrepancy in contemporary China[32].

Historically the rural-labor-exporting provinces, such as Henan, Shaanxi, Sichuan, Hubei, and Hunan, located in the Yellow River and the Yangtse River basins in central and western China[33], are the cradle to ancient Chinese agricultural culture, hence, migrant workers were born and bred up in traditional culture. In American anthropology, culture is defined as “the distinct ways that people, who live differently, classified and represented their experiences, and acted creatively”[34]. It generally includes everything that a group of people think, say, do and make, its customs, language, material artifacts and shared systems of attitudes and feelings[35]. As proposed by Jiang and Zhang[31, 36], agricultural and industrial cultures are divergent in many basic cultural elements, such as mode of economic production, lifestyles, dwelling conditions, common knowledge, social norms, beliefs, values, morals, law, folkways, and habits acquired as a member of society. Consequently, when migrant workers, the vehicles of agricultural culture, move to urban areas, the two distinct cultures come into contact and interacts. As a minority group in metropolitan cities, and a population with “weak culture” confronting the “strong culture” prevailing in a large city, migrant workers’ acculturation is not always a two-way interaction, but most often a hard process of one-way and passive adaptation to industrial culture[37].

By nature, culture is an integrated system consisting of at least four subsystems: material, institution, custom, and value[34]. At material level, the daily living of farmers rely on the soil, thus their means of livelihood are usually obtained through agricultural production. In contrast, urban society is a commercial society, the material means of subsistence of urban residents are derived from industrial production. The working experiences of migrant workers from agriculture possibly could not satisfy the requirements of modern industrial society and the cultural clash directly translates to ability to obtain necessary materials for a basic standard of living. At the level of institution, the social order of urban society is mainly maintained by law, regulations, and registration, while the maintenance of social order of rural villages is mainly dependent on the patriarchal clan system, blood relationship and Confucianism[38]. In other words, the rural community is an “acquaintance” or “relationship” society, but the urban community is a “stranger” society[39]. In this case, the second challenge for migrant workers is the breaking of social ties and interpersonal relationships based on clans. With regard to the differences in customs, rural and urban people are radically different in terms of etiquette, manners, festival folk customs, eating habits, wedding customs, religious ceremony, funeral systems, and the ceremony of sacrificing ancestors[40, 41]. For example, the implied rules of lining up for food, transportation and many other daily necessities that urban residents naturally comply with in their daily collective actions are alien habits to the newcomers from rural areas. Migrant workers from rural area are faced with the daunting task of revamping their daily habits from moment to moment. Lastly, value is the most crucial element of culture[35]. Daoism, Buddhism, Confucianism, and even Shanmanism are deeply rooted in migrant workers’ beliefs, and generally, Chinese farmers are always labeled as “diligent, hard-working, honest, and kind-hearted”[40]. However, the industrial society operates on market values, rather than human values. Migrant workers are thus challenged in their traditional value system when making complex moral decisions.

Although Chinese migrant worker population has experienced a remarkable demographic transition during the past three decades, for example, the new-generation migrant workers (born after 1980) had accounted for 45.1% of all migrant workers in 2014 [2], cultural characteristics of the new-generation are still similar to the old-generation, because new-generation migrant workers are generally brought up by their peasant parents/grandparents and culture can be transmitted and leaned from generation to generation [42]. Further, the past 30 years are also marked by striking cultural conflict between agricultural and industrial cultures, because the number of migrant workers had increased dramatically from 0 to 274 million during the same period [2] and the cultural contacts between rural and urban people should be very frequent. Given the increasing trend in number of migrant workers in the following years, the massive rural-to-urban migration in contemporary China provides a unique opportunity to examine rural-to-urban acculturative stress.

The discrepancies between rural and urban cultures so far discussed apparently has face validity but the intuitively assumed acculturative stress under the internal migration context is still inadequately addressed in research. Thus, the aim of this study is to explore the forms and contexts of acculturative stress among Chinese migrant workers.

## Materials and Methods

### Ethics Statement

Participants were fully informed of the study’s objectives, content, and procedures, and principles of confidentiality. Subjects who were migrant workers signed a written informed consent form before the interview. Because medical professionals involved in this study were only asked to discuss their experiences in providing mental health services to migrant workers and this procedure does not cause any harm or damage to them, participating medical professionals were only required to give oral consent prior to the interview. Although the use of written informed consent was waived for medical professionals, we still provided them with a statement describing the purpose and procedures of the study before they agreed to participate. The study protocol and the above-mentioned consent procedures received approval from the Survey and Behavioral Research Ethics Committee of the Chinese University of Hong Kong.

### Subjects

The present study was conducted in four factories of Shenzhen, China, between April and May 2012. Shenzhen is the largest migrant city of China with 7.8 million migrants from various parts of China in 2012, which accounted for 74.4% of its total population[43]. Qualitative data were collected from focus group discussions (FGDs) among migrant workers and individual interviews with frontline medical professionals who provided direct mental health care for workers of the selected factories and served within a community mental health service network funded by the local government. We chose migrant workers who were employed in factories because they accounted for over 80% of the migrant population in Shenzhen [5].

Researchers contacted local factory owners/managers through the local mental health service provider (Shenzhen Kangning Hospital) for permission to conduct the interviews in their promises. Migrant workers are eligible to participate in the study if they are at least 18 years old, originated from rural areas, working in factories, and living in Shenzhen without Shenzhen permanent ***hukou***. For a selected factory, the research assistant randomly selected several migrant workers from the list of workers that was provided by the factory, and invited them one by one, until four to five eligible migrant workers agreed to participate in our study. A total of 23 migrant workers were invited, six rejected to join the study because they were very busy. Participant characteristics of the 17 migrant workers who joined the four FGDs are shown in Table 1. In all, seven participants were men and 10 were women. Nine migrated from provinces of the Yangtse River basin and four from provinces of the Yellow River basin.

**Table 1:** Socio-demographic and migration-related characteristics of subjects of Focus Group Discussions (FGDs).

Round No. of FGD	Gender	Age (years)	Education	Marital status	Have children	Length of migration (years)	Age at first migration (years)	Province of rural origin	Types of industry	Monthly income (USD)	Frequency of returning hometown
1	Female	31	Junior high school	Married	Yes	14.0	17.0	Guangdong	Footwear	248–331	Once a month
	Female	32	Senior high school	Remarried	No	10.7	19.0	Hubei	Footwear	413–496	Once every two years
	Male	36	Junior high school	Never married	Yes	8.0	20.0	Chongqing	Footwear	248–331	Once a year
	Female	22	Senior high school	Never married	No	1.7	19.0	Hunan	Footwear	331–413	Once a year
2	Male	36	Senior high school	Married	Yes	17.0	19.0	Hunan	Electronics	496–579	Once a year
	Female	27	Senior high school	Married	Yes	7.0	19.0	Shaanxi	Electronics	331–413	Once a year
	Female	20	Junior high school	Never married	No	1.2	18.0	Guangdong	Electronics	331–413	Once a year
	Male	31	Illiteracy	Married	Yes	10.0	19.0	Guangdong	Electronics	413–496	Once a year
3	Male	30	Junior college	Separated	Yes	8.4	22.0	Hunan	Metallurgical	496–579	Once a year
	Female	23	Junior high school	Never married	No	4.3	18.0	Henan	Metallurgical	661–744	Once a year
	Female	20	Senior high school	Never married	No	0.5	18.0	Chongqing	Metallurgical	331–413	Once a year
	Male	24	Junior high school	Never married	No	1.2	16.0	Hubei	Metallurgical	413–496	Once a year
	Female	20	Senior high school	Never married	No	1.9	19.0	Guizhou	Metallurgical	331–413	Once every two years
4	Male	38	Senior high school	Married	Yes	12.2	24.0	Guangxi	Chemical	248–331	Once a month
	Male	39	Junior high school	Married	Yes	14.3	18.0	Shaanxi	Chemical	331–413	Once every three years
	Female	22	Senior high school	Never married	No	1.8	20.0	Hubei	Chemical	331–413	Once a year
	Female	23	Senior high school	Never married	No	2.0	19.0	Henan	Chemical	331–413	Once a year

Of the four selected factories, three had medical professionals who were providing mental health services. The three professionals, two men and one woman, were counselors with basic training in mental health assessment and psychological intervention and one to five years of experience in psychological consultation. They all agreed to join the individual interview. Their ages were 39, 44 and 47 years, respectively.

### Procedures

Interview topic guides were developed for the FGDs and for the individual interviews (Table 2), including, but not limited to, reasons for migration, perceived differences between cities and home villages, adaptation to urban lifestyle, connections with rural relatives and friends, relationship with city-dwellers, experiences of discrimination, housing conditions, working conditions, environment of workplaces, job arrangements and benefits, financial difficulties, and other problems encountered at the destination, as well as their own perspectives of life. FGDs and individual interviews were conducted in the meeting rooms of local factories or general practitioners’ offices of community health centers, which provided a convenient place with privacy for participants. All FGDs and individual interviews were conducted by a trained PhD student, with one research assistant taking detailed notes. All group discussions and interviews were audio-taped and fully transcribed. Each FGD took 60 to 90 minutes, while each individual interview lasted for 30 to 60 minutes.

**Table 2:** Guiding questions for the focus group discussion and individual interview^*^.

Core question 1	Would you please briefly talk about your (*their*) experiences of out-migration for work?
Probe 1	What is your (*their*) job before migration?
Probe 2	How about your (*their*) financial situation before migration?
Probe 3	Can you describe your (*their*) reason for leaving hometown to work in cities?
Probe 4	How long have you been in Shenzhen, how many cities you (*they*) have been to and how many jobs you (*they*) have done before?
Core question 2	Can you tell me about your (*their*) daily life in cities?
Probe 1	At the very beginning when you arrived at cities, what was your impression about cities?
Probe 2	What is the difference between rural and urban life?
Probe 3	Are there any obvious differences between villages and cities, in terms of environment, food, custom and interpersonal relationship? Please give some examples.
Probe 4	Facing these differences, how do you (*they*) adjust to the new life in cities?
Probe 5	What are your (*their*) main challenges in adapting to the new life in cities?
Probe 6	How do you (*they*) get along with urban workers and urban residents?
Probe 7	Did you (*they*) have any experiences of being looked down upon by urban people? If yes, please describe your (*their*) experience.
Probe 8	Would you please describe your (*their*) current living condition?
Probe 9	What do you (*they*) do at your (*their*) spare time?
Core question 3	Many families have “skeletons in the closet”. How about your (*their*) family?
Probe 1	How often do you (*they*) contact your (*their*) family and friends?
Probe 2	How about your (*their*) parents, wife or girlfriend, and children? Are they healthy? Is it difficult for your (*their*) children to attend school?
Probe 3	What is the financial situation of your (*their*) family? How many members of your (*their*) family have a paid job?
Probe 4	When you (*they*) get sick, in what condition you (*they*) will see the doctor?
Core question 4	How do you (*they*) define your current identity in cities?
Probe 1	How long will you (*they*) plan to stay in the city? If you (*they*) want to stay permanently, can you (*they*) achieve this goal successfully?
Probe 2	Compared to the rural people of your (*their*) origin, can you tell me some differences between you (*them*) and them (*rural people*), i.e., lifestyle, eating habits, and way of thinking?
Probe 3	Do urban people have any prejudice on you (*them*)? If yes, please give me at least two examples.
Core question 5	Can you describe your (*their*) current job?
Probe 1	How much is your (*their*) income? Are you (*they*) satisfied?
Probe 2	What social welfare benefits does your (*their*) job cover?
Probe 3	What is the environment of your (*their*) workplace like?
Probe 4	How good are the relationships that you (*they*) have with your (*their*) co-workers?
Probe 5	What are the disciplines against you (*them*) at work?
Probe 6	How to get a promotion in your (*their*) factory?
Core question 6	Would you like to add anything else?

*Questions for the focus group discussion and individual interview are nearly the same. Italics in parentheses are used for the individual interview.

### Data Analysis

Data analysis was conducted in a trial version of QSR NVivo 8 (QSR International Pty Ltd, Melbourne, Australia) using thematic content analysis[44]. First, we repeatedly read transcripts and notes to identify distinct text units, grouped and regrouped similar and dissimilar units, and generated initial categories. Next, we carefully went through all meaning units belonged to each category and re-distributed units and re-labeled categories as appropriate. As well, these categories were collapsed or subdivided as appropriate. Third, these coded transcripts were brought together into thematic categories that were exhaustive, mutually exclusive, conceptually clear and sensitizing. We repeated the procedures above until we were satisfied that these themes reflected the transcripts as a whole.

## Results

Nearly all study participants (16/17) admitted that their primary goal of out-migration for working in cities was to earn money, and the majority (15/17) reported being better off economically compared with their previous situation in rural hometowns. Six migrant workers said that they had adapted in cities, but 15 believed that Shenzhen was not a good place for permanent settlement, and 12 had plans to return to their hometowns. When asked about stress and difficulties related to the migration, all respondents, including the medical professionals, were able to describe many aspects which they perceived or observed as stressful or difficult for migrant workers. Five main themes emerged from the analysis of qualitative data: difficulties in adapting to the city environment, work-related stress, family-related stress, financial hardship and lack of sense of belonging to cities.

### Difficulties in adapting to the city environment

Since all subjects grew up in the quiet and natural environment of rural areas, most (14/17) complained of the noise, busy traffic, crowded streets, polluted air, and strange flavor of disinfected tap water. Those were particularly unacceptable during the early stage of their migration. They felt more difficult to adapt to the city environment. Three interviewees even ascribed their current medical conditions (insomnia, rhinitis, and stomach illness) to the polluted urban environment including noise, polluted air, and the “dirty” running water with a bleaching powder smell. As one respondent stated,

“*You know*, *after my arrival in Shenzhen*, *I often sneezed*, *and even sometimes had a runny nose*. *Initially*, *I thought what I just got was a cold*, *but this condition happened frequently*, *even more severe on days with heavier air pollution*. *Unfortunately*, *the doctor told me that I had an allergic rhinitis and needed to receive long-term medication treatment*. *I felt depressed*, *as I did not have such disease prior to my arrival*.”

### Work-related stress

Work-related stress was recognized as a major difficulty by all migrant workers. As a result of their low level of education, limited work experience and specific skills, most of the migrant workers were employed in labor-intensive industries. Sixteen respondents said that, they had to work overtime, get up early and go to bed late. Working 27–28 days per month and 12–14 hours per day was not uncommon. Sixteen interviewees complained about the extremely high workload and extended work hours. Work environment with latent health hazard was identified as another main source of work-related stress for all the 17 migrant workers. Working night shift was further recognized as complicating their daily life. The discipline at the four factories was also reportedly very strict by the 17 migrant workers; anyone who violated the regulations, made minor mistakes or could not complete assigned output quota, would therefore result in loss of bonus salary. Ten participants said that their supervisors were very harsh on workers and even strictly controlled workers’ time spent in washroom. If the time exceeded a pre-defined limit (usually five minutes), the violators would be penalized. In the words of a participant,

“*I have to arrive 15 minutes before our shift starts*, *to meet up for check-in*. *If I arrive five minutes late*, *I will be fined one-hour’s salary*. *We are not allowed to speak to others at work*. *The supervisors yell at us in public if we make mistake*. *I have been a front-line worker for five years and have never been so tired*. *Even if my father was hospitalized due to serious illness*, *and I need to ask for leave to my hometown*, *I will have to find our group supervisor*, *unit director*, *unit manager*, *and human resource department manager*, *one by one*, *to sign the note for leave*. *If you are not approved*, *but you really need to go back*, *the only choice is to voluntarily withdraw*. *What’s wrong with talking to others*? *What’s wrong with going back home to see my father*? *I can not work like a robot*.”

Two of the medical professionals also expressed their concerns for the possible adverse effects of migrant workers’ excessive workload. As one medical professional said:

“*These migrant workers are very busy*, *and often work under high pressure*. *On the one hand*, *they should work very fast to satisfy their production quota*. *On the other hand*, *they should be careful to avoid making mistakes*. *Several of my clients told me that they could not endure such high-demand job*. *They were overtired*, *and so they were stressed*.”

### Family-related stress

Because of the high urban living costs in cities, some migrant workers, who are parents of pre-school and school-aged children, and/or sons and daughters of their old parents, have to leave their children and/or parents in their rural hometowns. The separation between migrant workers and their family members, often induce frequent anxiety and feeling of guilt. All the 12 young parents in our study believed that the separation was harmful to their kids and/or old parents. Seven of them reported their uncontrollable worries about their children’s academic performance and all the 17 workers expressed concerns over their parents’ health. One migrant worker parent showed her worries as follows:

“*Now*, *I’m out for working in distant cities*, *with my husband*, *and we are lucky that we have two cute sons*. *However*, *we can’t live with them in Shenzhen*, *because they can’t be admitted to public schools to receive high-quality education and the tuition fees for private schools are too expensive for us*. *In my hometown*, *their grandparents are now living with them*, *and helping us raise them*, *but both grandparents are not very healthy*. *What should I do when one day I find that my parents also need to be taken care of*? *What should I do if my children acquire bad habits without parental monitoring*? *I sometimes feel nervous and lonely and I miss my family very much*. *I would be relaxed only if I was seated with them*. *I even can’t easily find a suitable time to talk to them on the phone*. *If I don’t need to earn money to support my family*, *I would not be so nervous like this*.”

In addition to the worries concerning the left-behind children and elders, a 20-year-old single young woman also shared her feelings of homesickness:

“*I have three younger brothers at school in my hometown and I have not been home for nearly three years*. *I have not played with them for a long time; even I don’t know what they’ve become*. *I miss them*, *my parents*, *and the little dog of my home*.”

### Financial hardship

Financial difficulties were reported to be the main reason why migrant workers had emigrated from rural lands, and seemed to continue to be a major source of stress after migration. Participants complained much more about their low wages (17/17), high living expenses (17/17), heavy economic burden to support parents and children (7/17), and expensive house rent (6/17). Other issues surrounding financial problems included excess expenditure in case of disease (11/17), insufficient pension after retirement (5/17), no other benefits except for wages (15/17), and no source of income in case of unemployment (13/17). One female migrant worker said:

“*I have very heavy financial burden that you can’t imagine*! *I’m the only person who has a paid job to support the whole family*. *My parents are farming in my village*, *and my two younger brothers and one younger sister are receiving education in schools*. *They all need money from me*. *Their living and schooling expenditures all rely on my slender income*. *Every month*, *when I receive their calls for money*, *I feel very anxious and helpless*, *as I have no more money to give them*, *I only retain 35USD for each month’s meal*. *For many years*, *I have not bought a new piece of clothing for myself*.*”*

All the medical professionals agreed that financial hardship was one of the leading stressors for migrant workers. The female medical professional said:

“*You have to know*, *migrant workers*, *who are married and middle-aged*, *the so-called* *“sandwich generation”*, *have many family members to support*, *including the parents-in-law*, *parents*, *and children*. *Thus*, *these workers are at higher risk for psychological problems*.”

### Lack of sense of belonging to cities

The loss of familiar social contact in a strange urban society emerged as another issue of concern. Many respondents felt “unsafe in cities” (11/17), said “should leave the city one day” (14/17), had “no friends, lonely” (15/17), believed they were “temporary people of the city” (16/17), and stated “there is no home here” (14/17). Fourteen interviewees mentioned difficulties when making friends with urban counterparts and other migrant workers, who were reported not to be interested in friendships but mainly focused on money and work. As one male migrant worker, who had migrated for work for many years and did more than 20 jobs in 19 cities, described:

“*For many*, *many years*, *in many*, *many cities*, *I work*, *I get fired*, *I start a new job again*, *and I get fired again…*. *I*, *look like someone loses his heart*, *work here*, *work there*, *come here*, *and go there; the only constant companion is the bag I have been carrying for years*. *I never know when there is an end to my wandering*.”

Nine participants complained that they were required to apply for Temporary Residence Permits (TRPs) if they are ready to work and live long in Shenzhen. They believed such requirement was unfair:

“*It is a joke*! *I’m a Chinese citizen from the rural area*, *and the city residents are also Chinese citizens*. *We are living and working in the same territory*, *we are all Chinese*. *Why only I should apply for the residence permit*?”

When migrant workers were asked about their social ties with local urban residents and colleagues, 15 interviewees reported that they had little dealings with them, except for work-related matters, because most of the time they were working and living in factories. One middle-aged man, who was living in a rented flat nearby the factory, described:

“*I have no local dweller friends*, *all my friends here are the acquaintances from my village*. *The only urban person*, *whom I frequently contact with*, *is the landlord*, *but I just have a tenancy relationship with him*. *After work*, *what I can do are just watching TV and sleep; I seldom go outside for window-shopping*, *therefore my social connection with the urban residents is very limited*. *As a temporary worker in the city*, *I believe* *“the less contacts with local people the better”*“.

## Discussion

The qualitative data in the present study revealed that Chinese migrant workers were confronted with a variety of sources of stress in their process of migration, including environment, occupation, family affairs, economic burden, and psychological sense of belonging. Overall, these findings on sources of stress are similar to those reported in sociological, anthropological and public health studies regarding disadvantages or difficulties of migrant workers [45–48]. However, our study is the first application of acculturative stress theory to understand stress associated with rural-to-urban acculturation in China and worldwide, because, as we proposed above, acculturative stress has been well studied among the Hispanic/Mexican and Asian immigrants in the United States [16,17]. Underlying causes of many challenges in the transition from rural-to-urban acculturation of Chinese migrant workers are detailed below.

In Chinese society, the traditional culture emphasizes more on “reverence for nature” and “people should live in harmony with nature”[49]. Modern urban culture alters the nature and created plenty of man-made devices, which contribute to comfort and convenience of modern lifestyle. However, the imbalance between modern technology and nature creates polluted air, water, busy traffic, and crowded environment that are so commonly intolerable to young migrant workers. Therefore, the material level of acculturation distress, as indicated by the first theme, difficulties in adapting to the city environment, seems inevitable and is experienced in everyday life.

The second theme, work-related stress, identified in this study, mainly reflects the conflicts between two types of economic productivity styles: agricultural and industrial productions. In traditional farming production, the Chinese farmers have been accustomed to working and resting as sunrise and sunset at a more natural and slower pace than the urban counterparts. Indeed, there is a high job demand for migrant workers employed in industry, but they only have very limited prior work experience that is largely from farming. The speed of modernized industrial production line creates an enduring daytime stress in work hours. Such work habit is perpetuated by at an institutional level. For instance, the industrial culture highly values efficiency, cost control and mass production at the expense of individual differences. The strict discipline on the migrant workers imposed by the factory’s managers and supervisors mirrors such values, which contrast starkly with the basic assumptions held by farmers in rural China and elsewhere in the world that productivity are not controlled by human discipline but largely “at the mercy of the force of nature”.

In the context of a traditional agricultural culture, Chinese people have an ingrained sense of family and they believe the well-being of all family members is more important than individuals[50]. The third theme, family-related stress, verified migrant workers’ deep sense of identification with family. The core principles of Confucianism in traditional Chinese culture consist of “loyalty”, “filial piety”, “benevolence”, and “righteousness”[51]. The trans-generational passage of Confucious principles are rooted in rural families where parents and grandparents taught the virtues of “respect and support of seniors”, “obedience to the will of the parents”, and “loving their families’ children”. At the institutional and custom level of acculturation, if a migrant worker’s father is dead without his/her care, he/she would be labeled as “violator of filial principles or disloyal to the family”. Similarly, under the guiding principles of Confucianism, financially supporting the elderly and children of their individual families is also categorized to be the inherent responsibility of migrant workers. When the migrant workers encounter lots of financial difficulties in cities, they still have to shoulder the family burden. On the contrary, modern urban culture advocates individualism, which means personal needs have priority over in-group needs[52]. This phenomenon could be regarded as an example of rural-to-urban acculturation on values.

It is interesting to find that migrant workers had too many complaints about their poor economic conditions while they still admitted that their economic situation had been improved. The traditional production mode of the “man ploughing and woman weaving" in rural China is self-sufficient, at least, Chinese farmers can directly get staple food such as rice and wheat through farming[53]. In industry culture, workers can not be the direct consumers of goods they produced; all daily necessities of migrant workers need to be obtained through currency, the medium of commodity exchange. In the case that migrant workers’ basic living resources need to be bought from markets, migrant workers may have a strong sense of economic insecurity, even worry and anxiety, because their wages are generally low and they probably will have nothing to eat if they discontinue work. Unlike traditional Chinese farmers, at least, they seldom worry about getting hungry. Owing to the deep sense of financial insecurity derived from different ways of getting living resources, and the heavy economic burden of supporting rural families, migrant workers reported financial hardship as a main challenge of their urban life.

The Chinese migrant workers are faced with unresolved social and economic barriers imposed by China’s national household registration system. Due to the restriction of their rural ***hukou***, they have limited access to local resources enjoyed by their urban counterparts, including social welfare, public service, medical care assurance, and other social security benefits[3]. Despite the fact that their presence has been very vital to the economic well-being of urban residents, this institutional exclusion makes migrant workers fall into the underclass of city societies. The absence of institutional support further leads to great difficulty in migrant workers’ integration into the mainstream of urban societies because most of them categorized them as short-staying passers-by of cities, rather than hosts of cities. As shown in this study, many migrant workers felt lost, aimless, confused, and alone about their urban living, mainly because they were uncertain about their cultural identity and future. In developed countries, the cultural identity of an international immigrant is likely to change and that encourages a degree of belonging after the settling down in the new culture [54], but the transition of cultural identity seems like an insuperable barrier for Chinese migrant workers. Indeed, China’s migrant workers have successfully changed their identity from farmers to industrial workers in occupation, but the social transformation of their cultural identity from farmers to a real sense of workers is far from done: they are still in the transition state of the so-called “peasant-workers”, neither farmers, nor urban residents. As a result, this study identified the sense of unbelonging to the cities as a major stressor of migrant workers’ rural-to-urban acculturation.

Previous transnational acculturative stress theories conceptualize the acculturative stress from various dimensions: social[55], attitudinal[55], familial[55], environment[55], discrimination[28,55,56], language barriers[28,56], and homesickness[28,56]. Because migrant workers were internal migrants of China, language barriers were not recognized as significant acculturative stressors. In this study, social discrimination seemed not a stressful challenge for migrant workers, however, this does not mean migrant workers did not have any discriminatory experiences, for example, the ***hukou*** system is an overt discriminatory measure against migrant workers in the provision of public services. On the other hand, a general type of discrimination, being looked down upon by urban residents, was not endorsed by our subjects, mainly because they were living and working together in a relatively homogenous environment, i.e., factories. Although several participants raised their concerns about their rural ***hukou***, i.e., being required to apply for TRPs, we consider it more related to cultural identity, thus we finally classified ***hukou***-related discrimination into “lack sense of belonging to cities”. Another finding that is very insistent with previously identified domains of international migrants’ stress is work-related stress, which is primarily explained by different reasons of the two populations’ migration: migrant workers migrate for work while international migrants for permanent settlement. Apparently, both Chinese migrant workers and international migrants are confronted with environment related stress, however, their specific forms are quite distinct, as migrant workers had more complaints about “hard environment”, such as noise and air pollution, but international migrants focused on “soft environment”, such as loosening ties with countries of origin and difficulties in getting occupational promotion[55]. These phenomena clearly reflect the unique contexts of acculturative stress of Chinese rural-to-urban migrant workers.

There are some potential limitations in the present study. First, our results are based on four rounds of FGDs and three in-depth interviews conducted in factories of Shenzhen. Further, Shenzhen is mainly a city of migrants, while migrant workers are in the minority in other cities of China. As such, whether these results could be generalized to migrant workers working in service industry or other migrant workers of other cities is still questionable. Nevertheless, these findings shed light on understanding the rural-to-urban acculturative stress experienced by migrant workers. Second, the identified rural-to-urban acculturative stressors might be confounded by other forms of general stress due to the cross-sectional nature of this study, these identified stress needs to be further verified in longitudinal studies.

Although there are certain limitations, our findings suggest that the rural-to-urban migration in China is a challenging process of transition with significant acculturative stress, indicating rural-to-urban cross-cultural maladjustment may be a fundamental pathogenic mechanism of poor mental health of migrant workers. Due to the high unmet psychological needs and the very large number of migrant workers[57], and many negative effects associated with acculturative stress, mental health services for migrant workers, from a public health perspective, should give priority to dynamic evaluation of rural-to-urban acculturative stress and health education on stress management, and, when necessary, individual psychosocial assessment and treatment.
